# A Gut Signature of Microbiome, Bile Acid, and Quorum-Sensing Profiles Is Associated with *Helicobacter pylori* Infection and Disease Progression

**DOI:** 10.3390/microorganisms14040801

**Published:** 2026-04-01

**Authors:** Hyder Alikhan, Brian White, John D. Sterrett, Marina Farag, Christopher A. Lowry, Lamya’a M. Dawud, Thomas Judge, Lark Perez, Joshua DeSipio, Sangita Phadtare

**Affiliations:** 1 Department of Internal Medicine, Cooper University Health Care, Camden, NJ 08103, USA; alikhan-hyder@cooperhealth.edu; 2 Division of Gastroenterology and Hepatology, University of Pennsylvania, Philadelphia, PA 19104, USA; brian.white1@pennmedicine.upenn.edu; 3 Department of Integrative Physiology, University of Colorado Boulder, Boulder, CO 80309, USA; john.sterrett@colorado.edu (J.D.S.); christopher.lowry@colorado.edu (C.A.L.); lamyaa.dawud@colorado.edu (L.M.D.); 4 Cooper Medical School of Rowan University, Camden, NJ 08103, USA; mfarag@une.edu; 5 Department of Gastroenterology, Cooper University Health Care, Camden, NJ 08103, USA; judge-thomas@cooperhealth.edu (T.J.); desipio-joshua@cooperhealth.edu (J.D.); 6 Department of Chemistry and Biochemistry, Rowan University, Glassboro, NJ 08028, USA; perezla@rowan.edu

**Keywords:** *Helicobacter pylori*, gut microbiome, autoinducer-2/AI-2, bile acids

## Abstract

Recent evidence suggests that *Helicobacter pylori* can act outside stomach by influencing the gut microbiome. We hypothesize that interactions between *H. pylori* and the gut microbiome, and the resulting changes in the gut chemicals (bile acids and bacterial signaling molecules involved in quorum sensing, e.g., autoinducer-2/AI-2), influence pathogen survival, antibiotic response, and disease progression. Our gut microbiome, bile acid, and AI-2 analyses showed that *H. pylori* patients had decreased alpha diversity (*p* = 0.05), increased AI-2 concentration (*p* = 0.019), decreased taurine-conjugated bile acids, and increased unconjugated bile acids. A co-occurring module of *Prevotella*, *Holdemanella*, and *Subdoligranulum*, was higher in patients (*p* = 0.04) and relative abundance of *Allisonella* was positively associated with levels of unconjugated bile acids, chenodeoxycholic acid, and cholic acid (*p* = 0.05 and 0.02, respectively). Our study is the first to characterize the gut microbiome-metabolome signature (bile acids and AI-2) in *H. pylori* patients. Abundance of certain bacteria that deconjugate bile acids along with increased AI-2 possibly gives selective advantage for *H. pylori* growth, further reducing microbial diversity. Taurine-conjugated bile acids inhibit *H. pylori* growth. We propose a model describing interplay of these factors in *H. pylori* disease progression, suggesting therapeutic targets worth exploring with rising antibiotic resistance.

## 1. Introduction

*Helicobacter pylori* is a Gram-negative, flagellated, helical bacterium that infects about half of the world’s population and is associated with peptic ulcers, non-ulcer dyspepsia, and gastric cancer [1,2]. *H. pylori* infection can also increase colorectal cancer risk by influencing gut bacteria, as it can act outside of its natural habitat, the stomach. High rates of *H. pylori* infections are seen in areas with socioeconomic challenges and overcrowded living situations [3]. Professional society guidelines recommend treatment of *H. pylori* infection with quadruple therapy, which may reduce the risk of gastric cancer [4,5]. Pretreatment antimicrobial susceptibility testing is generally not available, and treatment regimens are often chosen empirically, which reduces the likelihood of successful eradication. A recent study by Megraud et al. highlighted the urgent need for surveillance for antibiotic resistance and search for novel treatment strategies against *H. pylori* [6]. Traditional first-line treatment regimens have been increasingly ineffective against *H. pylori* infection due to emerging antibiotic resistance and insensitivity, which is influenced by several environmental factors and patient attributes [7]. As both antibiotic resistance/sensitivity and host physiological environment contribute to eradication, the information gained from gut microbiome and metabolome profiles will be highly informative. Here we study interplay between three physiological aspects with respect to *H. pylori* infection, (i) gut microbiome, (ii) bile acids, and (iii) bacterial interspecies signaling through quorum sensing with autoinducer-2 (AI-2).

The gut microbiome consists of inherently present intestinal microbes, some of which are beneficial for our health. *H. pylori* is associated with alterations of the ecological niche inhabited by the gut microbiome [8,9]. These also alter distal fecal microbiota [10]. Changes in the gut microbiome may influence the outcome of *H. pylori* infection. Although *H. pylori* predominantly colonizes the gastric mucosa, it is associated with duodenitis [11]. It is commonly associated with histopathological duodenitis, including in cases of gastric metaplasia, i.e., following development of stomach-like tissues in the duodenum. It is considered a leading cause of duodenal ulcers. In one study involving 206 duodenal biopsies, after excluding cases with celiac disease, 76 of the remaining 198 patients had duodenal histopathological abnormality, while *H. pylori* was found in 95 (47.9%) of patients. Of patients with histopathological duodenitis, 59% were *H. pylori*-infected and the rate of duodenitis was higher in patients with *H. pylori* infection. Overall, these data support the conclusion that *H. pylori* is a major contributor to duodenitis in regions with high prevalence of *H. pylori* infections. In addition, there is recent growing evidence for *H*. *pylori* infection increasing colorectal cancer risk as this bacterium can act outside of its natural habitat, the stomach. It has been proposed that besides a potential direct effect, *H*. *pylori* may be exerting indirect effects through gut bacteria [12,13,14].

Bile acids are cholesterol-based molecules produced by the liver and carry out the emulsification of dietary fats. While the impact of *H. pylori* on the chemical profile of gut bile acids has not been characterized, there is significant evidence for strong interactions between *H. pylori* and bile acids. Human bile acids are shown to inhibit the growth of *H. pylori* in vitro [15]. Another in vitro study showed that conjugated bile acids act as chemorepellents for *H. pylori* [16]. As the chemorepellent properties are distinct for the chemically different bile acids and the chemical profile of bile acids present in the gut is impacted by the host and the microbiome, we postulate that the dissemination of *H. pylori* may also correlated with the concentrations of specific bile acids present in the gut.

Certain bacteria produce quorum sensing signaling molecules such as AI-2, which interact with other bacteria and potentiate signaling cascades that play a role in virulence of the host bacteria [17,18,19]. *H. pylori* can undergo drastic and rapid changes in cellular morphology and biofilm state. Many of these changes are induced through quorum sensing. *H. pylori* produces AI-2 [20]. *H. pylori* biofilm formation increases tolerance to higher oxygen levels, pH, temperature, nutrient levels, and antibiotics, suggesting that quorum sensing may play a critical role in its antibiotic resistance [21]. This notion is further supported by the involvement of AI-2 in the regulation of various aspects of the *H. pylori* life-cycle including flagellin expression and biofilm formation [20]. Therefore, we hypothesize that assessing AI-2 levels in *H. pylori* patients will inform the disease progression.

This is the first report of interplay between changes in gut microbiome profiles, gut bile acid profiles, and quorum sensing profiles in association with *H. pylori* infection studied by evaluating respective fecal profiles. Our overall objective is to characterize the chemical and microbiological patterns of gut dysbiosis associated with *H. pylori* infection. This information will help create alternate means to minimize these changes and formulate customized treatment regimens for its eradication.

## 2. Materials and Methods

### 2.1. Patient and Sample Collection

Our study recruited patients who presented to the Cooper University Hospital (CUH) in Camden, New Jersey. *H. pylori* patients (34) and healthy controls (26) at least 18 years of age were eligible for inclusion. *H. pylori* infection was confirmed via biopsy and control participants were recruited from a healthy population at CUH. Patients less than 18 years old, pregnant individuals, or those who were taking antibiotics for three months before and/or during sampling were excluded from participation. Informed consent was obtained by one of the team members on the study. Demographic data alongside relevant medical and clinical history were collected through surveys. Additional clinical data including lab values, diagnosis, medications, and comorbidities were collected using the electronic medical record system (EPIC). Patients were asked to provide a stool sample before starting eradication therapy. Stool samples were collected using the cotton swab method and immediately put on ice for transport prior to freezing at −80 °C. Gut microbiome, bile acids and quorum sensing analyses were carried out for each sample.

### 2.2. Analyses of Gut Microbiome, Bile Acids and AI-2

Total genomic bacterial DNA was extracted using the Qiagen DNeasy PowerSoil HTP Kit (Qiagen, Redwood City, CA, USA). NanoDrop spectrophotometry was used to quantify DNA before processing. High-throughput 16S rRNA gene amplicon sequencing was conducted as described previously [22]. For bile acid analysis, stool samples were lyophilized to remove excess water, weighed and analyzed using liquid chromatography-mass spectrometry (LC-MS) (Metabolon Inc., Durham, NC, USA) (additional details described in Appendix A.2). Fifteen bile acids were measured (Appendix B). The measured concentrations were corrected for weight in ng/g of sample. We standardized quorum sensing assays using human fecal samples; this is the first report of this kind. For these assays, Vibrio harveyi TL26 and (S)-4,5-dihydroxy-2,3-pentanedione (DPD) were used as the stable analog for autoinducer-2 (AI-2) [23]. Each sample was prepared and analyzed in quintuplet (additional details described in Appendix A.1).

### 2.3. Statistical Analyses

Sequencing data analysis was performed using Quantitative Insights Into Microbial Ecology 2 (Qiime2) 2020.11 and Linear Discriminant Analysis effect size (LEfSe) as described previously [22]. Sequences were de-multiplexed, filtered, and clustered into amplicon sequence variants (ASVs) using QIIME 2 DADA2. A naïve-Bayes classifier trained on the latest SILVA version 138 16S rRNA gene database (March 2021) was used to assign the taxonomy via the QIIME 2 interface. Additional Python packages 3.9 (SciPy, Statsmodels, Scikit-bio) were used for statistical tests on QIIME 2-generated data. Diversity analyses were performed in QIIME 2, rarefied to an even sampling depth of 36,000 reads per sample. Taxa were considered differentially abundant with LDA score > 2.0 and *p* < 0.05 based on two-tailed Kruskal–Wallis and Wilcoxon tests. Alpha diversity was assessed via Pielou’s evenness, Faith’s phylogenetic diversity (PD), observed features and Shannon diversity index via Kruskal–Wallis test for nominal variables or via Spearman correlation test for numerical variables. Unweighted UniFrac was considered the primary metric for beta diversity and PERMANOVA was used to assess group-based differences in microbiome community composition for beta diversity. Differences in taxa were assessed by analysis of compositions of microbiomes with bias corrections 2 (ANCOM-BC2, via Scikit-bio) [24]. The *p*-values from differential abundance testing were corrected for false discovery rate (FDR) using the Benjamini–Hochberg method. Sparse correlation network investigation for compositional data analysis was used to cluster taxa at the species level into highly co-occurring modules. We utilized Sparse Co-occurrence Network Investigation for compositional data (SCNIC) to conduct a co-occurrence network analysis. The SCNIC co-occurrence network analysis was performed following the procedure outlined by Shaffer et al. [25]. In summary, SparCC was used to calculate correlations between ASVs, and the shared minimum distance algorithm in SCNIC was used to cluster and sum the relative abundances of highly co-occurring ASVs into “modules”, using a minimum *R* value of 0.35. Differentially abundant modules were assessed using ANCOM-BC2 [26,27]. AI-2 concentrations were determined using AI-2 detecting, lux-producing bacteria. Bile acid concentrations were determined by liquid chromatography-mass spectrometry. AI-2 concentrations and bile acid concentrations were compared between the *H. pylori* patient group and the healthy control group using Student’s *t*-test (*p* < 0.05). The *p*-values for each demographic and clinical characteristic noted in Table 1 were calculated using Chi-squared (*χ*^2^) test.

### 2.4. Ethics Approval

This study was approved by the CUH IRB (17-077). All procedures performed in studies involving human participants were in accordance with the ethical standards of the institutional and/or national research committee and with the 1964 Helsinki Declaration and its later amendments or comparable ethical standards.

## 3. Results

Table 1 shows the demographic data for the study participants. Consistent with our previous observation [22], a majority of the *H. pylori* patients that participated in this study were females (60%), >40 years of age (82%), and African American or Hispanic (82%).

**Table 1:** Patient demographics and clinical characteristics.

	Total (*N* = 60)	*H. pylori* Patients (*n* = 34)	Controls (*n* = 26)	*p*-Value
Sex, *n* (%)				0.75
Male	24 (40)	13 (38)	11 (42)	
Female	36 (60)	21 (62)	15 (58)	
Age, *n* (%)				0.13
18–30	4 (7)	2 (6)	2 (8)	
31–40	7 (12)	2 (6)	5 (19)	
41–50	13 (22)	8 (24)	5 (19)	
51–60	23 (38)	11 (32)	12 (46)	
61–70	11 (18)	10 (29)	1 (4)	
71–80	2 (3)	1 (3)	1 (4)	
Race, *n* (%)				0.43
African American	16 (27)	11 (32)	5 (19)	
Asian American	4 (7)	2 (6)	2 (8)	
Caucasian	5 (8)	1 (3)	4 (15)	
Non-white Hispanic	33 (55)	19 (56)	14 (54)	
Other	2 (3)	1 (3)	1 (4)	
Smoker, *n* (%)	5 (8)	2 (6)	3 (12)	0.43
Past *H. pylori* infection, *n* (%)	9 (15)	5 (15)	4 (15)	0.94
Past *H. pylori* treatment, *n* (%)	6 (10)	2 (6)	4 (15)	0.05
Duodenal ulcer, *n* (%)	3 (5)	3 (9)	0 (0)	0.12
Non-ulcer dyspepsia, *n* (%)	35 (58)	21 (62)	14 (54)	0.54

We analyzed the association of each of the factors included in Table 1 with alpha diversity. Several factors showed statistically significant differences (*p* ≤ 0.05) in alpha diversity by observed features analysis. These include (i) smoking (*p* = 0.005), (ii) people per household (*p* = 0.003), (iii) past use of proton pump inhibitors (PPI) (*p* = 0.001), (iv) sex (*p* = 0.05), (v) current use of PPI (*p* = 0.05), (vi) duodenal ulcer (*p* = 0.04), (vii) non-ulcer dyspepsia (*p* = 0.03), and (viii) *H. pylori* infection (*p* = 0.05). Additionally, age also showed significant difference (*p* = 0.039) in alpha diversity in Pielou’s evenness analysis.

**Figure 1:**
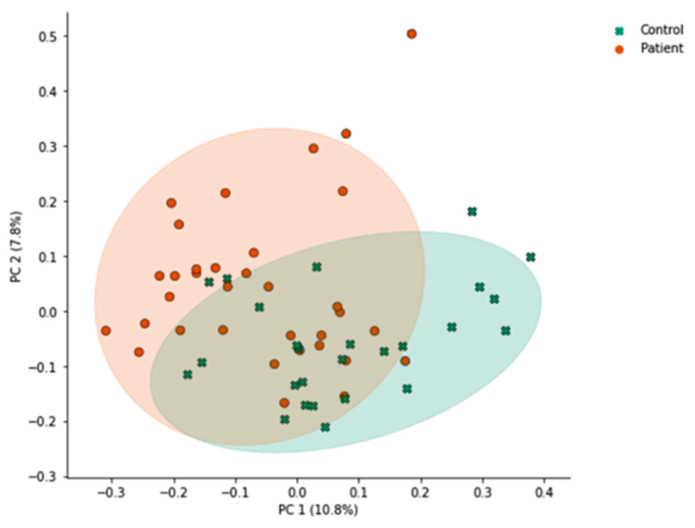
Microbiome beta diversity of healthy controls versus *H. pylori* patients. Unweighted UniFrac PCoA plots are shown comparing healthy control participants (green, *n* = 26) and *H. pylori* patient samples (orange, *n* = 34). Percentages along each axis show the portion of phylogenetic variance across samples captured by that axis. Each point represents the phylogenetic composition of one sample. Ellipses represent 95% confidence intervals of the group’s PCoA coordinates. Abbreviations: principal coordinates analysis axis (PC), principal coordinates analysis (PCoA).

Unweighted UniFrac principal coordinate analysis (PCoA) (Figure 1) shows ordination of healthy control participants and *H. pylori* patients. PCoA plots are shown comparing healthy control participants and *H. pylori* patient samples. Each point represents the phylogenetic composition of one sample, and shaded regions represent 95% confidence intervals of the first 2 PCoA axes for each group. Points that are close together have similar phylogenetic composition, and points that are far apart have dissimilar phylogenetic composition. The proportion of variance explained by each principal coordinate axis is denoted in the corresponding axis label; PC1 explains 10.8% of the variation and PC2 explains 7.8% of the variation across samples. *H. pylori* patients had significantly different overall community composition, compared to control subjects, based on unweighted UniFrac PERMANOVA (*p* = 0.001; Figure 1). While statistically significant, the proportion of variance explained by the first two axes is low (PC 1, 10.8%; PC 2, 7.8%; total, 18.6%), meaning that these axes represent a small percentage of the overall variability in the full dataset distributed across all dimensions included in the PERMANOVA. Additionally, ethnicity (*p* = 0.023), past use versus no past use (*p* = 0.008) and current use versus no current use (*p* = 0.036) of PPI, and non-ulcer dyspepsia (*p* = 0.05) were associated with differences in beta diversity as measured by unweighted UniFrac.

**Figure 2:**
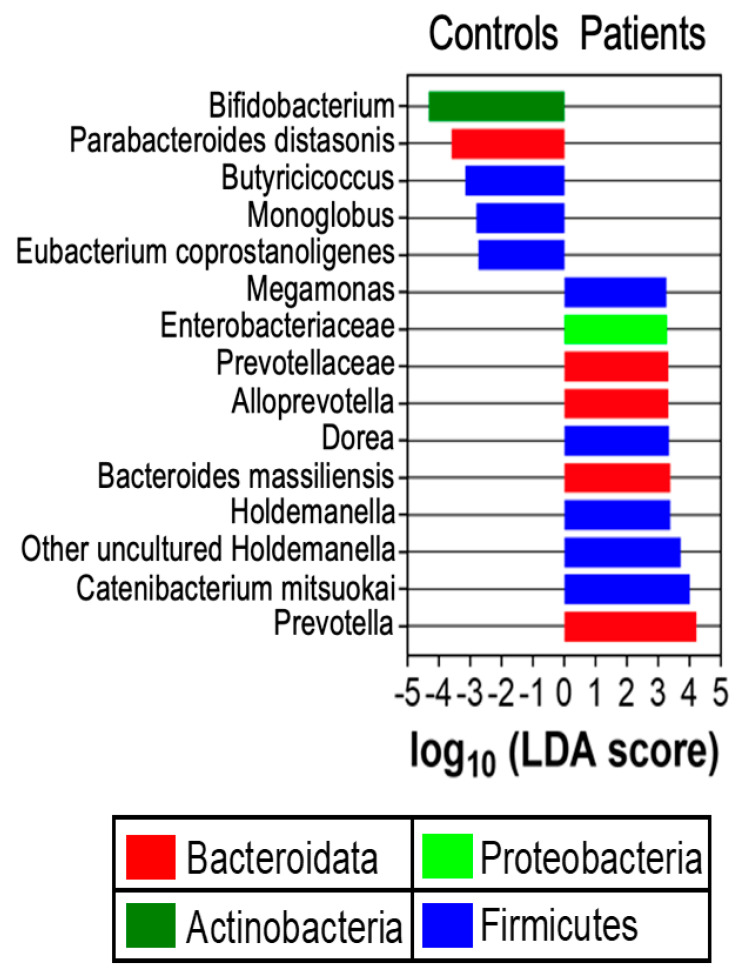
Linear discriminant analysis effect size (LEfSe). LEfSe scores are presented for differentially distributed taxa across *H. pylori* patients (*n* = 34) versus healthy control participants (*n* = 26) via Kruskal–Wallis and Wilcoxon tests with two-tailed α = 0.05. Negative and positive values represent taxa that were enriched in healthy control participants and *H. pylori* patients, respectively. Each phylum is represented by a different color.

Linear discriminant analysis effect size (LEfSe) analysis was carried out to determine differential relative abundance of bacterial taxa between *H. pylori* patients and healthy controls. LEfSe scores were differentially distributed between the two groups (Figure 2). *Prevotella*, *Bacteroidota*, *Proteobacteria*, and *Holdemanella* taxa had higher relative abundance in *H. pylori* patients compared to control participants (*p* = 0.04). Certain species within the *Bacteroides* genus were more abundant in patients compared to controls, for example, *Bacteroides massiliensis.*

We then performed a multivariable PERMANOVA (Appendix C) including smoking status, current PPI use, and prior *H. pylori* treatment as covariates to assess potential confounding. Differences in gut microbiome composition between *H. pylori* patients and controls remained significant after adjustment (*R*^2^ = 0.038, *p* = 0.0006), indicating that the observed microbiome signature is not explained by these clinical variables. PPI use was independently associated with microbiome variation (*R*^2^ = 0.045, *p* = 0.027), while smoking showed a non-significant trend and prior treatment history had no detectable effect. Furthermore, homogeneity of dispersion did not differ significantly between groups (betadisper ANOVA, *p* = 0.076), supporting that the PERMANOVA results reflect differences in community composition rather than dispersion. These findings demonstrate that the association between *H. pylori* status and the gut microbiome is robust to adjustment for key clinical confounders.

**Figure 3:**
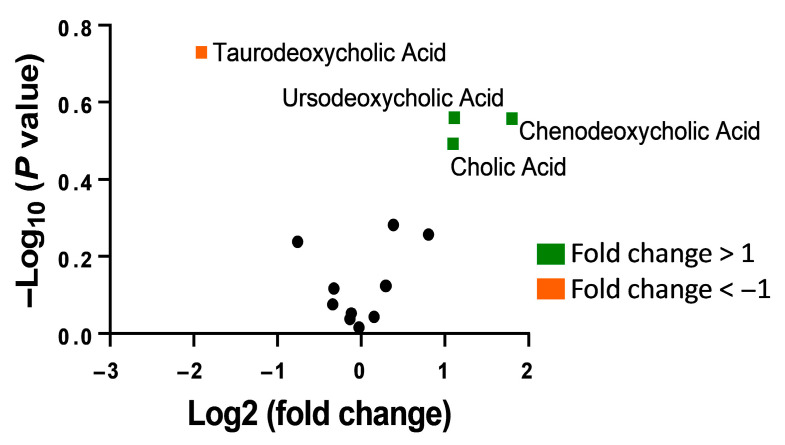
Volcano plot showing composition of bile acids in healthy control participants versus *H. pylori* patient groups.

Analysis was carried out to determine differences in bile acids between the two participant groups. As seen from the volcano plot presented in Figure 3, compared to healthy control participants, *H. pylori* patient samples showed an increase in unconjugated bile acids including cholic acid and chenodeoxycholic acid while a decrease was observed in the conjugated bile acid taurodeoxycholic acid.

**Figure 4:**
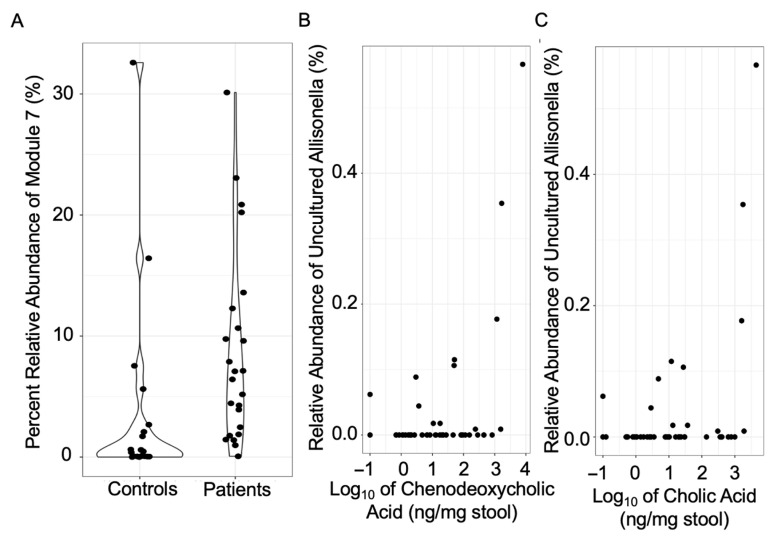
Gut microbiome and bile acids with respect to *H. pylori* infection. (**A**) A highly co-occurring module (module 7) of *Prevotella*, *Holdemanella*, and *Subdoligranulum* was identified as higher in relative abundance in *H. pylori* patients. (**B**,**C**) The relative abundance of an uncultured taxon within the genus *Allisonella*, which was positively associated with the concentration of chenodeoxycholic acid and cholic acid.

Next, we explored the correlation between bacterial taxa and bile acids that are influenced by *H. pylori* (Figure 4). ANCOM-BC2 uses pairwise log ratios to account for the compositionality of the data, and the output usually comprises only a few differentially abundant taxa. A highly co-occurring module (module 7) of *Prevotella*, *Holdemanella*, and *Subdoligranulum* was identified as higher in relative abundance in *H. pylori* patients (Figure 4A). The relative abundance of an uncultured taxon within *Allisonella* was positively associated with the level of chenodeoxycholic acid and cholic acid (*p* = 0.05 and 0.02, respectively) (Figure 4B,C). ANCOM-BC2 corrects for multiple comparisons by controlling the mixed directional false discovery rate (mdFDR) across taxa and, when using pairwise comparisons, across the different group pairs [28].

**Figure 5:**
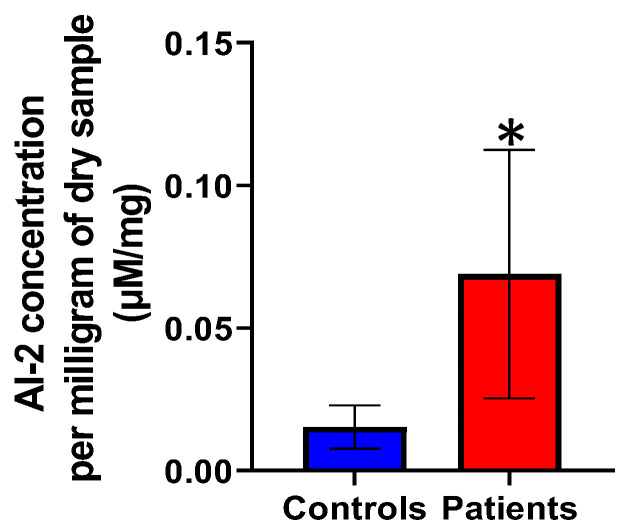
Comparison of the level of AI-2 in *H. pylori* patients and healthy control participants. Error bars represent 95% confidence intervals. * indicates statistical significance.

Autoinducer-2 (AI-2) concentrations per milligram of dry weight were compared between *H. pylori* patients and healthy controls as shown in Figure 5. AI-2 concentrations were statistically significantly higher in the *H. pylori* patient group than those in the healthy controls group (*p* = 0.019).

## 4. Discussion

The finding of higher relative abundances of *Bacteroidota* and *Proteobacteria* observed in *H. pylori* patients as compared to control participants in this study is consistent with previous observations from our and other groups [22,29]. Among these, higher relative abundance of a highly co-occurring module (module 7) of *Prevotella*, *Holdemanella*, and *Subdoligranulum* was observed via ANCOM-BC2 analysis. Our observations were consistent with the increase in *Prevotella* and *Holdemanella* seen in *H. pylori* patients by Frost et al. [10]. Increases in the relative abundance of *Prevotella* were also observed in the duodenal microbiome of *H. pylori* patients [30] and in *H. pylori* patients with advanced gastric lesions [29]. It was also reported that upward movement of the bile acids due to the duodenogastric reflux can alter the gut microbiome [31]. Increases in the relative abundance of a highly co-occurring module (module 7) including *Subdoligranulum* was observed in our *H. pylori* patients, including those without duodenogastric reflux.

Our study introduces the idea of bile acid characterization using human stool samples in *H. pylori* patients. We observed an increase in unconjugated bile acids including cholic acid and chenodeoxycholic acid in *H. pylori* patients as compared to healthy controls. This is consistent with previous studies that explored bile acid composition in in vitro models [15]. Unconjugated dihydroxy bile acids such as chenodeoxycholic acid, cholic acid, and ursodeoxycholic acid have no inhibitory effects on *H. pylori* [32]. We observed a decrease in taurine conjugated bile acids in *H. pylori* patients. Previous in vitro models indicate that glycine- and taurine-conjugated bile acids inhibit *H. pylori* growth [15]. Since conjugated bile acids also act as chemorepellents for *H. pylori* [16] and these are decreased in *H. pylori* patients, this may allow for ease of dissemination of *H. pylori* within the gut. This suggests interplay between bile acid conjugation and *H. pylori* disease progression.

Unconjugated bile acids like chenodeoxycholic acid have also been shown to increase human cell signaling through the FXR pathway in gastric cells in in vitro models that may lead to gastric intestinal metaplasia and gastric cancer [33,34], whereas, ursodeoxycholic acid has a more protective role in bile acid signaling pathways [35]. Because *H. pylori* is associated with elevated unconjugated bile acids like chenodeoxycholic acid and these bile acids can cause intestinal metaplasia and gastric cancers seen in *H. pylori* infection, bile acid dysregulation may be a possible mechanism through which *H. pylori* promotes carcinogenesis [36].

Interestingly, ANCOM-BC2 analysis showed that *Allisonella* is associated with unconjugated bile acids such as chenodeoxycholic acid and cholic acid. *Allisonella*, to our knowledge, has not previously been shown to alter bile acids.

Bacteria differ in their metabolism of bile acids. In particular, *Bacteroides* can deconjugate bile acids with bile salt hydrolase, especially taurine- and glycine-conjugated bile acids, and they can oxidize, epimerize, and esterify bile acids [35,37,38,39]. *Bacteroides* have also been implicated in *H. pylori* infection, as mentioned previously; certain species within the *Bacteroides* genus were more abundant, for example, *Bacteroides massiliensis* (Figure 2). There is a possibility that *Bacteroides* deconjugates bile acids that act as chemorepellents for and inhibit the growth of *H. pylori* suggesting interplay between *H. pylori* infection, gut microbiome composition, and bile acid composition.

To the best of our knowledge, detection of AI-2 signaling molecule in human stool samples for *H. pylori* infection is novel. Since *H. pylori* impacts distal fecal microbiome, AI-2 concentrations would be affected as well. We observed that *H. pylori* patients had statistically significantly higher AI-2 concentration as compared to healthy control participants. This was consistent with increased AI-2 concentrations observed in the in vitro models [40]. Certain virulence factors that are important for the *H. pylori* infection are upregulated with increases in AI-2 concentrations. These include increases in the urease expression that leads to decreases in the gastric acidity [41], and flagella expression that increases motility of *H. pylori* [20].

**Figure 6:**
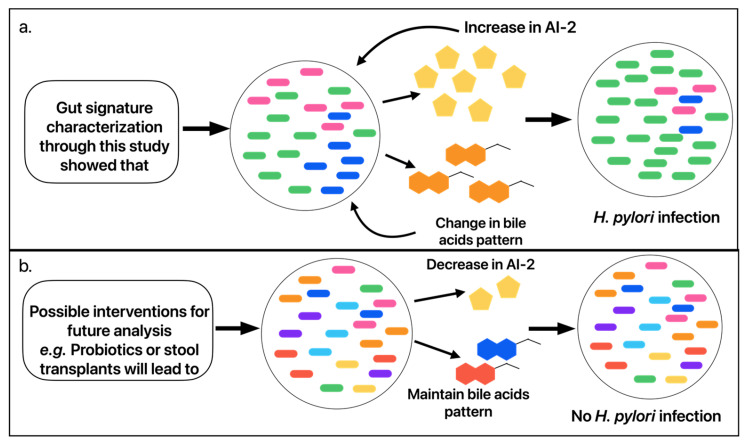
Schematic representation of potential implication of gut signature in *H. pylori* treatment. (**a**) High proportion of *H. pylori* (green oval) increases AI-2 production and causes possible alterations in bile acids, which leads to a favorable environment for *H. pylori* growth. This in turn increases the risk of sustained *H. pylori* infection. (**b**) Possible future interventions that maintain high abundances of beneficial gut bacteria. This will decrease the AI-2 production and prevent alterations in bile acids. This will lead to a less favorable environment for *H. pylori* growth, decreasing the risk of its sustained infection.

AI-2 analogs have the potential to manipulate and antagonize AI-2 quorum sensing signaling pathways [42]. These analogs could potentially inhibit *H. pylori* and other associated bacterial cascades. Autoinducer-2 is detected by *H. pylori*, but it can also be synthesized and detected by other bacteria as well. *Prevotella*, a genus that we have shown to be associated with *H. pylori*, has the ability to synthesize and detect AI-2 [43,44]. *Bacteroides*, another genus that we have shown to be associated with *H. pylori* infection and that deconjugates bile acids, also produces and detects AI-2 [45,46,47]. We present a model (Figure 6) that illustrates these relationships, in which *H. pylori* infection associates with higher relative abundance of specific bacterial taxa (i.e., *Prevotella* and *Bacteroides*) that associate with altered bile acid composition (i.e., *Bacteroides*-mediated deconjugation of bile acids inhibitory to *H. pylori* and those that act as chemorepellents) and increased bacterial quorum sensing signaling molecules (i.e., AI-2). However, it is uncertain if these gut microbiome and metabolome changes precede and facilitate *H. pylori* infection or if they result from *H. pylori* infection.

Strengths of the study include concurrent investigation of gut microbial diversity and community composition, bile acid composition, and quorum sensing pathways in patients with *H. pylori* infection and controls. Women were included in the study (60%), and the patient population spanned a broad range, from the 18–30 to the 70–80 age groups, improving generalizability. One of the limitations of the study is the relatively small sample size, which is contributed in part by the socioeconomic aspects of our subjects. The county area from which the subjects were recruited has the lowest median income and highest unemployment and poverty in the state. Significant fraction of our subject population is below the national poverty line [48]. In addition, the ethnic minority groups living this area suffer from significant socioeconomic disparities and low educational attainment. This may lead to patients’ lack of understanding of the significance of the study and their decreased inclination to participate. A large number of potential participants were not able to return to the clinic to return stool samples. This was because of the lack of access to suitable transportation or inability to leave home or work during the required timeframe for sample collection and delivery, which is after diagnosis but before they start antibiotics. Small sample size may affect power calculations. Due to power limitations, our interpretations are weakened by the lack of adjustment for clear and significant confounding variables; for example, alpha diversity is associated not only with *H. pylori* status (*p* = 0.05) but also with smoking, household size, sex, and past/current PPI use (*p*-values from 0.05 to 0.001). Similarly, beta diversity is associated with ethnicity and PPI use. Another limitation is that microbiome diversity and community composition, bile acid analysis and AI-2 analyses were conducted using fecal samples, which reflect the distal gut, not the primary gastric niche of *H. pylori*.

## 5. Conclusions

Our characterization of an *H. pylori* gut signature suggests that the interplay between the gut microbiome, bile acid, and quorum sensing pathways may affect *H. pylori* pathophysiology, influencing pathogen survival, antibiotic response and disease progression. Our observation of a potential role for the AI-2 signal in *H. pylori*’s unique microbiome-metabolome environment presents additional possible therapeutic targets worth exploring as antibiotic resistance is on the rise.

## Data Availability

We are now depositing de-identified data into Qiita, an open-access microbial study management platform, under study ID number 16454. We plan to submit this to the permanent data repository of the European Bioinformatics Institute (EBI). Analytical methods are described with sufficient details and references to allow reproduction.

## Abbreviations

The following abbreviations are used in this manuscript:

AI-2	Autoinducer-2
ANCOM-BC2	analysis of compositions of microbiomes with bias correction 2
*H. pylori*	*Helicobacter pylori*
LC-MS	Liquid Chromatography-Mass Spectrometry
LEfSe	Linear Discriminant Analysis effect size
Qiime2	Quantitative Insights Into Microbial Ecology 2
ASV	amplicon sequence variants
PPI	Proton Pump Inhibitor

## Appendix A

### Appendix A.1

**Autoinducer-2 bioluminescence assay using human stool samples.**

*V. harveyi* TL26 bioluminescent strain was kindly provided by Dr. Bonnie Bassler, Princeton University, USA. The strain does not produce its own AI-2. The strain was cultured in Lysogeny Broth (LB) medium. (*S*)-4,5-dihydroxy-2,3-pentanedione (DPD) was purchased from Sigma-Aldrich (St. Louis, MO, USA). The assays were performed in flat clear bottom black polystyrene 96-well plates. Stool samples were collected on swabs. Deionized, sterile water (2 mL) was added to the swab collection tube and vortexed at 13,000 r.p.m. The solution was then transferred to sterile conical tubes and centrifuged at 13,000 r.p.m. for 2 min. The supernatant was filtered through a sterile 0.22-micron filter to obtain the final processed stool samples. Overnight culture of *V. harveyi* TL26 was diluted in fresh LB medium 1:100 and then added to the assay tubes. DPD was added with a starting concentration of 1.5 μM and diluted serially 3-fold. Patient and control processed stool samples were added to the assay tubes at a 1:10 dilution. The assay mixtures were incubated at 30 °C in a plate reader, with shaking capability, for 20 h. AI-2 signaling activity was quantified using lux detection followed by OD_600_ absorbance measurement every 15 min. OD_600_ growth curves and DPD dose dependent curves using Relative Light Units (RLU) were generated in GraphPad Prism 9.4.0 (GraphPad Software, Inc., Boston, MA, USA). RLU was determined using lux and OD600 measurements. Patient and control sample AI-2 levels were interpolated using Graphpad Prism and the DPD dose dependent curves for each assay. The assays were carried out in quintuplets.

### Appendix A.2

**Measurement of bile acids in human fecal samples.**

*H. pylori* patient and healthy control processed samples were lyophilized to remove any excess water. The mass of each sample was measured, and bile acids were analyzed by liquid chromatography mass spectrometry. The measured concentrations were corrected for weight in ng/g of sample. The quantification of bile acids was performed by Metabolon, Inc. (Morrisville, NC, USA). Briefly, freeze-dried human feces samples were stored at −80 °C until analysis. Bile acid concentrations were analyzed by LC-MS/MS (Metabolon Method TAM178: “LC-MS/MS Method for the Quantitation of Bile Acids”). Calibration ranges for the individual analytes ranged from 0.25 ng/mL to 250 ng/mL. Calibration samples were prepared at eight different concentration levels by spiking an acidified methanol solution with corresponding calibration spiking solutions. Calibration samples, study samples, and quality control samples were spiked with a solution of labeled internal standards and subjected to protein precipitation using acidified methanol. Following centrifugation, an aliquot of the organic supernatant was evaporated to dryness using a gentle stream of nitrogen. The dried extracts were reconstituted and injected onto an Agilent 1290 Infinity/SCIEXQTRAP 6500 LC-MS/MS system equipped with a C18 reverse phase UHPLC column (Agilent, Santa Clara, CA, USA). The mass spectrometer was operated in negative mode using electrospray ionization (ESI). The peak area of each bile acid parent (pseudo-MRM mode) or product ion was measured against the peak area of the respective internal standard parent (pseudo-MRM mode) or product ion. Quantitation was performed using a weighted linear least squares regression analysis generated from fortified calibration standards prepared immediately prior to each run. LC-MS/MS raw data were collected using SCIEX software Analyst 1.7.3 and were processed using SCIEXOS-MQ v.1.7. Data reduction was performed using Microsoft Excel for Office 365 v.16.

## Appendix B

**Table A1:** Quantified bile acids.

Bile Acid	Log 2 (Fold Change)	−Log (*p*-Value)
Chenodeoxycholic Acid	1.80222649	0.557335139
Cholic Acid	1.099953456	0.492137973
Deoxycholic Acid	−0.027105589	0.015488926
Glycochenodeoxycholic Acid	0.290271759	0.123664819
Glycocholic Acid	0.154945074	0.043046683
Glycodeoxycholic Acid	0.804019296	0.257072912
Glycolithocholic Acid	0.2998	0.123569352
Glycoursodeoxycholic Acid	−0.117191165	0.052512075
Lithocholic Acid	0.386025193	0.281739056
Taurochenodeoxycholic Acid	−0.339469632	0.075573675
Taurocholic Acid	−0.759897076	0.238368207
Taurodeoxycholic Acid	−1.90834087	0.729232422
Taurolithocholic Acid	−0.325671395	0.116682727
Tauroursodeoxycholic Acid	−0.133361833	0.037407392
Ursodeoxycholic Acid	1.112547849	0.559406012

## Appendix C

https://github.com/Sangita-pixel/H-pylori-gut-signature (accessed on 24 March 2026)
